# GRF1 fine‐tunes the copper‐responsive transcription module of CPK3‐bHLH107 by manipulating interaction and phosphorylation

**DOI:** 10.1111/tpj.71150

**Published:** 2026-09-30

**Authors:** Yue Yu, Haoran Xia, Ran Dong, Haifeng Liu, Xiangsong Chen, Zhaohui Chu

**Affiliations:** ^1^ State Key Laboratory of Hybrid Rice, Hubei Provincial Research Center for Basic Biological Sciences, College of Life Sciences Wuhan University Wuhan 430072 China; ^2^ Hubei Hongshan Laboratory Wuhan 430070 China; ^3^ Shandong Institute of Pomology Tai'an 271000 China; ^4^ College of Agronomy Shandong Agricultural University Taian 271018 China

**Keywords:** *ACS8*, bHLH107, copper ion, CPK3, plant immunity, phosphorylation, 14‐3‐3

## Abstract

Previously, the crucial role of calcium‐dependent protein kinase 3 (CPK3) in Cu^2+^‐triggered immunity (CuTI) has been established in Arabidopsis. CPK3 promotes the phosphorylation of basic helix–loop–helix 107 (bHLH107), thereby activating the expression of *1‐aminocyclopropanecarboxylic acid synthesis 8* (*ACS8*) and downstream defense responses. However, the precise mechanisms underlying the fine‐tuning of CPK3‐mediated phosphorylation of bHLH107 remain unclear. To elucidate the mechanism underlying the inactivation of the CPK3‐bHLH107 module, we identified a 14‐3‐3 protein, GRF1 (GF14χ), among candidate bHLH107 interactors. Notably, the interaction between GRF1 and bHLH107 was weaker following Cu^2+^ treatment than under control conditions. In *grf1* mutant plants, the transcript level of *ACS8* was slightly increased, concomitant with a modest increase in resistance to *Pst* DC3000. GRF1 interacts with both CPK3 and bHLH107 to maintain an ‘OFF’ state. Cu^2+^ mediates the phosphorylation of CPK3, attenuating its interaction with GRF1 and promoting its dissociation from GRF1. Subsequently, CPK3 phosphorylates bHLH107 and GRF1, thereby switching the immune response to the ‘ON’ state. Furthermore, wild‐type GRF1, the quadruple phospho‐defective mutant GRF1^S72/88/125/156A^ and the quadruple phosphomimetic mutant GRF1 ^S72/88/125/156D^ directly interact with unphosphorylated bHLH107, but not with the double phosphomimetic mutant protein bHLH107^S62/72D^. We also revealed that GRF1 attenuates CPK3 kinase activity. Collectively, our findings indicated that CuTI is precisely orchestrated through GRF1‐mediated modulation of the CPK3‐bHLH107 module in Arabidopsis.

## Significance Statement

The protein interaction and phosphorylation of the CPK3‐bHLH107 module are essential for copper‐triggered immunity; however, the mechanisms governing its activation and repression remain uncharacterized. Here we revealed the role of GRF1 in fine‐tuning the CPK3‐bHLH107 module. GRF1 interacts with CPK3 and unphosphorylated bHLH107, but not with phosphorylated bHLH107 substrates, thereby modulating immune status. Furthermore, GRF1 attenuates CPK3 kinase activity.

## INTRODUCTION

Copper (Cu^2+^) plays two sides of the coin in soil: it serves as an essential mineral element required for plant growth and development, yet can also act as a toxic heavy metal contaminant when accumulated in excess. Moreover, Cu^2+^ contributes significantly to plant protection against a broad spectrum of microorganisms as a component of copper‐based antimicrobial compounds (CBACs), which are widely employed as bactericides and fungicides (Lamichhane et al., 2018). Cu^2+^ has been used commercially for approximately 140 years and has recently been shown to trigger immunity in plants, termed copper‐triggered immunity (CuTI), thereby establishing double‐ or triple‐tiered protection (Lamichhane et al., 2018; Liu et al., 2015, 2020; Yu et al., 2023). Foliar application of Cu^2+^ promotes calcium influx, rapid ethylene bursts, and the accumulation of salicylic acid (SA), further stimulating various plant immune responses (Liu et al., 2015, 2020; Yu et al., 2021; Zhang et al., 2018). Furthermore, supplementation of nutrient solution with Cu^2+^ to mimic root treatment has been shown to enhance antiviral and antifungal resistance in rice (Chen et al., 2025; Yao et al., 2022). Regardless of whether Cu^2+^ is applied to roots or leaves, CuTI relies mainly on the transport of Cu^2+^ into leaf cells at infection sites via plasma membrane‐localized copper transporters (COPTs) (Xia et al., 2026; Yao et al., 2022). Additional Cu^2+^ might be transported via plasma heavy metal transporters (Chen et al., 2025). However, the perception and signal transduction mechanisms underlying Cu^2+^‐triggered immunity remain poorly understood.

*Arabidopsis thaliana* encodes thirteen distinct 14‐3‐3 isoforms, designated omega (ω), psi (ψ), chi (χ), phi (φ), upsilon (υ), pi (π), omicron (ο), nu (ν), mu (μ), lambda (λ), kappa (κ), iota (ι), and epsilon (ε) (Sedlov & Sluchanko, 2025). These 14‐3‐3 proteins (14‐3‐3 s) are highly conserved and interact with a wide range of partner proteins to regulate plant metabolism, hormone signaling, stomatal movement, and stress response (Chen & Qiu, 2022; Dong et al., 2023; Huang et al., 2018; Lachaud et al., 2013; Tseng et al., 2012; Zhao et al., 2021). Emerging evidence indicates that 14‐3‐3 s function as hubs that collaborate with or inhibit the function of numerous kinases by modulating their translocation, stability, or kinase activity through reversible phosphorylation events (Fan et al., 2023; Gao et al., 2022; Lachaud et al., 2013; Yang et al., 2019). However, the role of 14‐3‐3 s in regulating the kinase activity of calcium‐dependent protein kinases (CPKs) remains unclear. GRF1 (GF14χ) is a 14‐3‐3 protein localized in both the cytoplasm and nucleus and has been implicated in seed development (Swatek et al., 2011, 2014). Previous studies have demonstrated that shade light induces the dephosphorylation and nuclear accumulation of PIF7, and that blocking the interaction between PIF7 and 14‐3‐3 proteins (GF14λ/κ) accelerates shade‐triggered dephosphorylation and nuclear localization of PIF7 (Huang et al., 2018). Notably, PIF7 can also interact with GRF1; however, whether GRF1 participates in the regulation of PIF7 remains unclear (Huang et al., 2018).

Plant innate immune systems comprise pathogen‐associated molecular pattern (PAMP)‐triggered immunity (PTI) and effector‐triggered immunity (ETI), both of which utilize Ca^2+^ influx and reactive oxygen species (ROS) for signaling. External‐stimuli‐induced Ca^2+^ influx is usually controlled by Ca^2+^‐permeable channels, with Ca^2+^‐binding proteins responsible for transmitting Ca^2+^ signals (Ren et al., 2023; Weralupitiya et al., 2024). As one of calcium‐binding proteins, CPKs play critical roles in plant development, immunity, and stress response (Yip Delormel & Boudsocq, 2019). In particular, *Arabidopsis thaliana* CALCIUM‐DEPENDENT PROTEIN KINASE 3 (*CPK3*) functions as a signal transducer and controls diverse physiological processes, such as stomatal movement, abiotic and biotic stress responses, seed germination, and pathogen defense, through the phosphorylation of various substrates (Arimura & Sawasaki, 2010; Li, Hu, et al., 2025; Li, Wang, et al., 2025; Lu et al., 2020; Mehlmer et al., 2010; Ming et al., 2025; Schulze et al., 2021; Wu et al., 2013). Cold stress rapidly activates CPK3, which is essential for the early activation of CYCLIC‐NUCLEOTIDE‐GATED CHANNEL 5 (CNGC5) and CNGC6 during the initial stage of calcium signaling (Ming et al., 2025). CPK3 plays a vital role in decoding calcium signals by phosphorylating SnRK2s (Li, Hu, et al., 2025). Besides, CPK3 may mediate ethylene‐induced H_2_O_2_ formation in guard cells through its interaction with RBOHD and RBOHF (Li, Wang, et al., 2025). In addition, CPK3 positively regulates Arabidopsis resistance against *Pseudomonas syringae pv. tomato* (*Pst*) DC3000 by phosphorylating actin‐depolymerization factor 4 (ADF4) (Lu et al., 2020). Notably, previous studies have shown that CPK3 phosphorylates GRF1 at the Ser72, Ser88, Ser125 and Ser156 residues (Swatek et al., 2014); however, the molecular mechanism by which the CPK3‐GRF1 complex regulates immunity in Arabidopsis remains unclear.

Basic helix–loop–helix (bHLH) 107 belongs to the bHLH transcription factor family, one of the largest transcription factor (TF) families in plants (Carretero‐Paulet et al., 2010). Our previous studies revealed that Cu^2+^ activates CPK3 in Arabidopsis leaf, which subsequently phosphorylates its interacting substrate bHLH107 to activate the expression of the targeted gene *1‐aminocyclopropanecarboxylic acid synthesis 8* (*ACS8*) and enhance Arabidopsis resistance to *Pst* DC3000 (Yu et al., 2026). Here, we report that the 14‐3‐3 protein GRF1 acts as a molecular switch that controls CPK3 kinase activity and the transcription ability of bHLH107. Cu^2+^ rapidly stimulates CPK3 phosphorylation, which is essential for bHLH107 phosphorylation, disrupts the interaction of GRF1 with both CPK3 and bHLH107, and consequently activates *ACS8* expression and downstream defense responses. These findings advance our understanding of CuTI signal transduction and provide broader insight into 14‐3‐3 s‐CPKs regulatory interactions in plants.

## RESULTS

### GRF1 interacts with both bHLH107 and CPK3 and functions in Cu^2+^‐triggered immunity

Previously, we revealed that the CPK3‐bHLH107 phosphorylation complex positively regulates Cu^2+^‐mediated resistance in Arabidopsis (Yu et al., 2026). Given that 14‐3‐3 s are well‐established scaffolds in CPK‐mediated phosphohorylation (Ormancey et al., 2017), we sought to identify potential switches for regulating Cu^2+^‐triggered phosphorylation signals within the CPK3‐bHLH107 module. To this end, we remined previously immunoprecipitation‐mass spectrometry (IP‐MS) data using bHLH107 as bait (Yu et al., 2026), derived from 4‐week‐old Arabidopsis seedlings treated with or without 100 μM CuSO_4_ for 2 h. Among the candidate bHLH107 interactors, two 14‐3‐3 proteins, GRF1 and GRF5 (GF14υ), exhibited reduced interactions with bHLH107 following Cu^2+^ treatment (Figure 1A, Figure S1A). These findings suggest that GRF1 and GRF5 may function to restrain CPK3‐bHLH107 phosphorylation triggered by Cu^2+^. To validate the interaction between bHLH107 and selected 14‐3‐3 s (GRF1 and GRF5), we performed pull‐down assays (Figure S1B). We found that bHLH107 directly interact with GRF1 *in vitro*, whereas no direct interaction was detected with GRF5 (Figure S1B). Furthermore, we obtained a *grf5* T‐DNA insertion mutant (SALK_014690.37.25.x) and examined the transcript level of *ACS8*. The results showed that, similar to Col‐0, Cu^2+^‐induced *ACS8* expression was unaffected in the *grf5* mutant (Figure S1C). Collectively, these data indicate that GRF5 may not be involved in Cu^2+^‐induced defense response.

**Figure 1:**
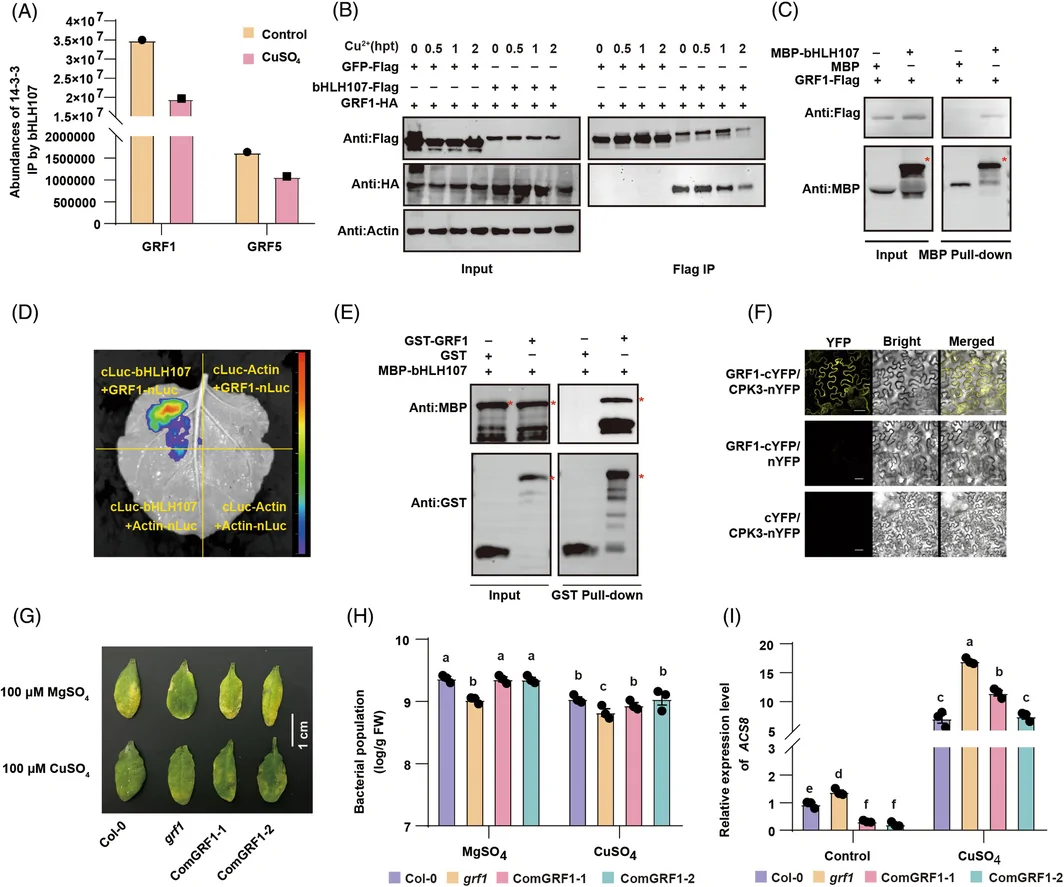
GRF1 interacts with both bHLH107 and CPK3 and functions in Cu^2+^‐triggered immunity. (A) Abundances of GRF1 and GRF5 captured by bHLH107 in the presence or absence of CuSO_4_ treatment, as determined by IP‐MS. (B) Co‐IP assay was performed to analyze the intensity of interaction between GRF1 and bHLH107. Samples were harvested at 0, 0.5, 1, and 2 h after Cu^2+^ treatment. GFP‐Flag was coexpressed with GRF1‐HA as the negative control. (C–E) The interaction between GRF1 and bHLH107 was performed with *semi‐in vivo* co‐IP (C), LCA (D), and pull‐down (E) assays. For co‐IP, *N. benthamiana* transiently expressing GRF1‐Flag was incubated with prokaryotically expressed MBP‐bHLH107, as shown in Figure 1C. For LCA, transient coexpression of cLuc‐bHLH107 with GRF1‐nLuc was performed, and cLuc‐bHLH107 or GRF1‐nLuc was coexpressed with Actin‐nLuc (LOC_Os01g05490) or cLuc‐Actin as the negative control, as shown in Figure 1D. For pull‐down assays, prokaryotically expressed GST‐GRF1 and GST immunoprecipitated MBP‐bHLH107 *in vitro*, and the precipitated proteins were analyzed by immunoblotting with anti‐GST and anti‐MBP antibodies, as shown in Figure 1E. (F) The interaction between GRF1 and CPK3 was evaluated using BiFC assays. Transient coexpression of CPK3‐nYFP with GRF1‐cYFP in *N. benthamiana*. Coexpression of CPK3‐nYFP with cYFP, or GRF1‐cYFP with nYFP, served as negative controls, as shown in Figure 1F. (G–I) Disease symptom images (G), bacterial load of *Pst* DC3000 (H), and relative *ACS8* expression (I) in wild‐type Col‐0, *grf1*, and two independent homozygous complemented lines (ComGRF1‐1 and ComGRF1‐2). Four‐week‐old plants were sprayed with CuSO_4_ (100 μM) or MgSO_4_ (100 μM) and inoculated with *Pst* DC3000 4 h later. The bacterial populations were quantified at 3 days post‐inoculation (dpi) (G‐H). For qRT‐PCR, the samples were harvested at 2 h after treated with or without CuSO_4_. Expression levels were normalized to *Actin2* (*At3g18780*). Error bars represent the means ± SEMs (*n* = 3). Asterisks indicate significant differences compared with the control (Student's *t*‐test) (I).

To further confirm that Cu^2+^ impairs the interaction between GRF1 and bHLH107, we performed a luciferase complementation assay (LCA) and observed that Cu^2+^ treatment partially attenuated the GRF1‐bHLH107 interaction (Figure S1D,E). We subsequently verified this reduced interaction using a co‐immunoprecipitation (co‐IP) assay in *Nicotiana benthamiana* (*N. benthamiana*) (Figure 1B). Based on these results, we selected GRF1 for further investigation of its role in regulating the Cu^2+^‐triggered phosphorylation signaling within the CPK3‐bHLH107 module.

To verify the authenticity of the GRF1–bHLH107 interaction, we performed a *semi‐in vivo* co‐IP assay, which demonstrated that MBP‐tagged bHLH107, but not MBP alone, interacts with Flag‐tagged GRF1 (Figure 1C). We further validated this interaction using an LCA in *N. benthamiana* (Figure 1D) and a pull‐down assay (Figure 1E). Notably, it has been reported that CPK kinase forms a dimer with 14‐3‐3 s to regulate downstream signaling pathways (Fan et al., 2023). We further demonstrated that GRF1 interacted with CPK3 using a bimolecular fluorescence complementation (BiFC) assay (Figure 1F). Overall, these results indicate that GRF1 physically interacts with both bHLH107 and CPK3.

To further investigate whether the complexes of GRF1‐bHLH107 and GRF1‐CPK3 function within the Cu^2+^‐response immunity module, we assessed the bacterial growth of *Pst* DC3000 in wild‐type Col‐0, *grf1* (SALK_018664C), and two independent homozygous complementation lines expressing *ProGRF1::gGRF1‐Flag* in the *grf1* background (ComGRF1‐1, ComGRF1‐2). Compared with Col‐0, the *grf1* mutant exhibited slightly reduced *Pst* DC3000 growth. Both the susceptibility and Cu^2+^‐mediated resistance to *Pst* DC3000 were recovered in the ComGRF1‐1 and ComGRF1‐2 lines relative to *grf1* (Figure 1G,H), indicating that *GRF1* is involved in Cu^2+^‐mediated resistance against *Pst* DC3000. Furthermore, under untreated conditions, the basal expression level of *ACS8* was slightly increased in *grf1* but reduced in ComGRF1‐1 and ComGRF1‐2 compared with the wild‐type (Figure 1I). Notably, Cu^2+^‐induced *ACS8* expression was markedly higher in *grf1* than in Col‐0, ComGRF1‐1, and ComGRF1‐2 (Figure 1I). These findings suggest that GRF1 negatively regulates *ACS8* expression under normal conditions but is not required for bHLH107‐mediated Cu^2+^‐driven upregulation of *ACS8*. Given that Cu^2+^ activates the phosphorylation of CPK3 and bHLH107 (Yu et al., 2026), these results strongly imply that GRF1 may function as an inhibitor of the CPK3‐bHLH107 module in the Cu^2+^‐responsive immune pathway.

### Cu^2+^ disrupts the CPK3‐GRF1 complex

GRF1 interacts with CPK3 and bHLH107, and loss of GRF1 enhances Cu^2+^‐induced upregulation of *ACS8*. Therefore, we speculated that Cu^2+^‐mediated phosphorylation of CPK3 or bHLH107 attenuated their interaction with GRF1. A previous study identified four phosphorylation sites on GRF1‐Ser^72^, Ser^88^, Ser^125^ and Thr^156^‐ that are phosphorylated by CPK3/28 (Swatek et al., 2014). However, the role of these phosphorylation sites in Cu^2+^ signaling has not been explored. We first examined whether CPK3 interacts with GRF1 and its phospho‐mutants. BiFC assays demonstrated that CPK3 interacted with wild‐type GRF1, the quadruple phospho‐defective mutant protein GRF1^S72/88/125/156A^ (GRF1‐4A), the quadruple phosphomimetic mutant protein GRF1^S72/88/125/156D^ (GRF1‐4D) (Figure 2A). These interactions were further confirmed by co‐IP assays in *N. benthamiana* (Figure 2B). Given that Cu^2+^ treatment impaired the bHLH107–GRF1 interaction (Figure 1A,B, Figure S1D,E), we hypothesized that Cu^2+^ may also promote dissociation of the GRF1‐CPK3 complex. To test this, we performed co‐IP experiments to assess the effect of Cu^2+^ treatment on the GRF1–CPK3 interaction. Compared with the untreated control, the interaction between GRF1 and CPK3 was significantly reduced following Cu^2+^ treatment (Figure 2C). We also examined the GRF1 transcript levels in Col‐0 following treatment with 10 μM or 100 μM CuSO_4_ for 0, 0.5, 1, and 2 h. The results showed that *GRF1* expression showed no significant alteration in response to either concentrations of CuSO_4_ (Figure S2C). We further assessed GRF1 protein levels in the two complementary lines (*ComGRF1‐1* and *ComGRF1‐2*), as well as in *N. benthamiana* leaves transiently expressing *GRF1* under the control of its native promoter, following treatment with different concentrations of CuSO_4_. Our results indicated that CuSO_4_ treatment did not appreciably induce GRF1 protein degradation (Figure 2D). Taken together, these findings demonstrate that Cu^2+^ promotes the dissociation of the CPK3‐GRF1 complex.

**Figure 2:**
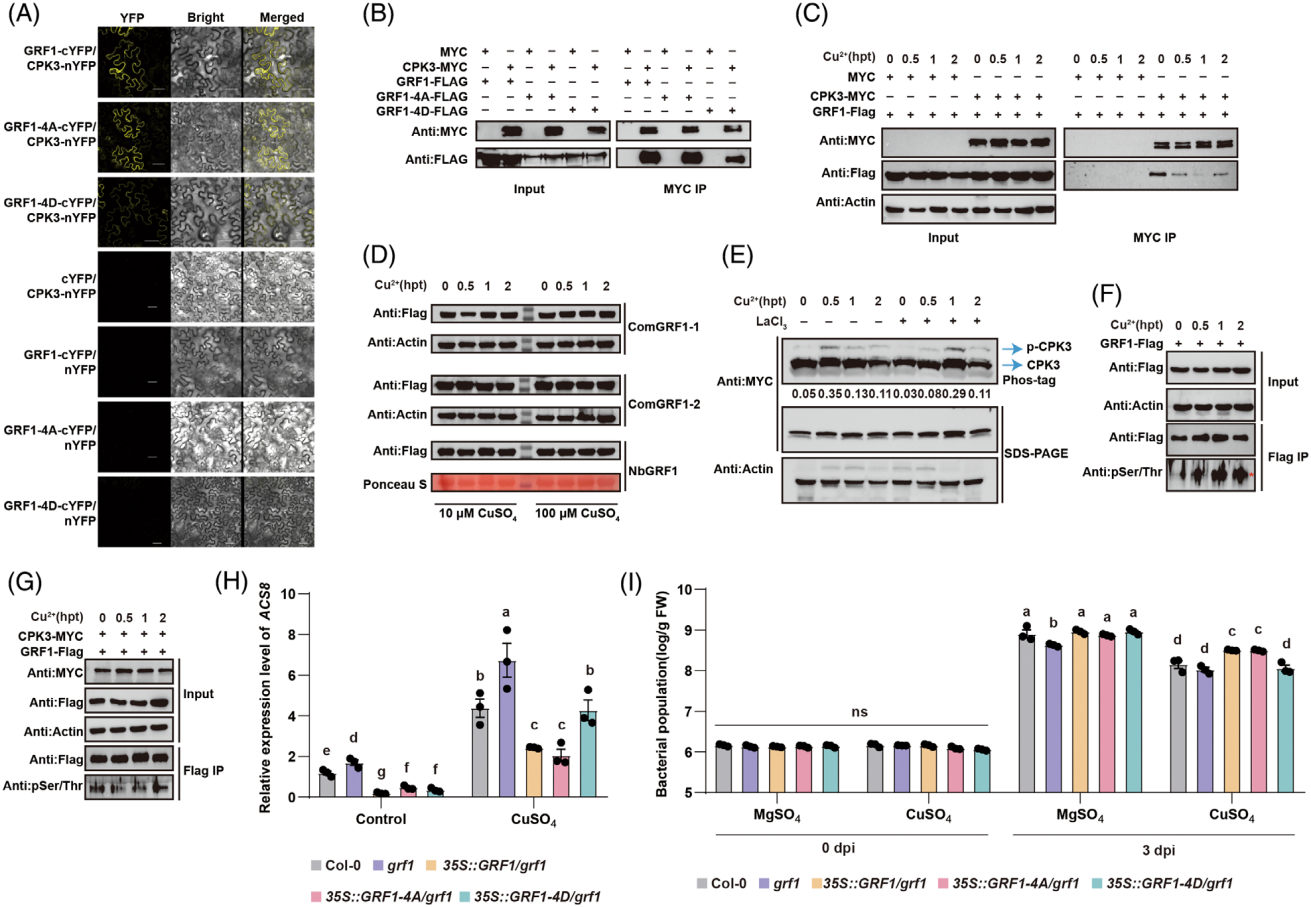
Cu^2+^ disrupts the CPK3‐GRF1 complex. (A, B) Interactions of GRF1, GRF1‐4A, and GRF1‐4D with CPK3 were determined by BiFC (A) and co‐IP (B) assays. The methods used were previously described in Figure 1. (C) Co‐IP assay analyzing the interaction between GRF1 and CPK3. Samples were harvested at 0, 0.5, 1, and 2 h after Cu^2+^ treatment, and *35S::MYC* was coexpressed with *35S::GRF1‐Flag* as the negative control. (D) Protein level of GRF1 in Arabidopsis ComGRF1‐1, ComGRF1‐2, and in *N. benthamiana* transiently expressing *ProGRF1::GRF1*. (E) Cu^2+^‐induced phosphorylation of CPK3 following pretreatment with 10 mM LaCl_3_, CPK3‐MYC was transiently expressed in *N. benthamiana*. CPK3 and phosphorylated CPK3 (p‐CPK3) are marked with blue arrows. (F, G) *In vivo* kinase assays showing CPK3‐mediated phosphorylation of GRF1. Expressing GRF1‐Flag in *N. benthamiana* for 60 h and spraying with CuSO_4_ (100 μM), the samples were harvested at 0, 0.5, 1, and 2 h for analysis (F). For the experiment shown in (G), CPK3‐MYC and GRF1‐Flag were coexpressed in *N. benthamiana* for 60 h, followed by treatment with CuSO_4_ (100 μM), the samples were harvested at 0, 0.5, 1, and 2 h for analysis. (H, I) Relative *ACS8* expression (H) and bacterial load of *Pst* DC3000 (I) in wild‐type Col‐0, *grf1*, *35S::GRF1/grf1*, *35S::GRF1‐4A/grf1*, and *35S::GRF1‐4D/grf1*. The methods used are described in Figure 1.

We previously revealed that Cu^2+^‐activated CPK3 phosphorylation and Ca^2+^ signaling play important roles in positively regulating CuTI (Yu et al., 2026). To determine whether this process is dependent on Ca^2+^ signaling, we examined Cu^2+^‐induced phosphorylation of CPK3‐MYC following pretreatment with the Ca^2+^‐channel blocker LaCl_3_. Compared with the untreated control, CPK3 phosphorylation response was delayed in the LaCl_3_‐pretreated group; it did not reach its highest level at 0.5 h compared to control, but instead peaked at 1 h (Figure 2E). These results indicated that Cu^2+^‐triggered CPK3 phosphorylation is partially dependent on Ca^2+^ signaling. Additionally, to determine whether Cu^2+^‐triggered CPK3 phosphorylates GRF1, we examined Cu^2+^‐induced phosphorylation of GRF1 transiently expressed in *N. benthamiana* following treatment with 100 μM CuSO_4_ for 0, 0.5, 1, and 2 h. As shown in Figure 2F, Cu^2+^ induced phosphorylation of GRF1 (Figure 2F). We further coexpressed GRF1‐Flag with CPK3‐MYC and treated with 100 μM CuSO_4_, the results indicated that Cu^2+^ specifically activates CPK3 to phosphorylate GRF1 (Figure 2G).

To investigate the specific role of GRF1‐4A and GRF1‐4D in the Cu^2+^‐induced defense response, we generated *35S::GRF1‐Flag*, *35S::GRF1‐4A‐Flag* and *35S::GRF1‐4D‐Flag* constructs and introduced them into the *grf1* background (*35S::GRF1/grf1*, *35S::GRF1‐4A/grf1*, and *35S::GRF1‐4D/grf1*), respectively (Figure S2A). We examined the relative transcript levels of *ACS8* in these plants after CuSO_4_ treatment and found that *ACS8* expression in *35S::GRF1/grf1* and *35S::GRF1‐4A/grf1* was lower than that in Col‐0 (Figure 2H). Furthermore, no significant difference was observed between Col‐0 and *35S::GRF1‐4D/grf1* under Cu^2+^ treatment (Figure 2H). We further measured the bacterial population at 3 days post‐inoculation (dpi). Compared with Col‐0, *35S::GRF1/grf1* and *35S::GRF1‐4A/grf1* plants exhibited slightly increased susceptibility to *Pst* DC3000, whereas the susceptibility of *35S::GRF1‐4D/grf1* was not significantly different with Col‐0 plants (Figure 2I). Collectively, these results demonstrate that Cu^2+^‐mediated phosphorylation of CPK3 disrupts its interaction with GRF1 through a phosphorylation‐dependent mechanism.

### GRF1 impaired CPK3 kinase activity

To determine the effect of GRF1 on the CPK3–bHLH107 interaction, we performed an LCA in which CPK3‐nLuc and cLuc‐bHLH107 were transiently coexpressed with either 35S::*GRF1*‐*Flag* or the *35S::Flag* empty vector (PL43_PS1300‐Flag) as a negative control. Compared with the control, coexpression of GRF1 with CPK3‐nLuc and cLuc‐bHLH107 significantly reduced the fluorescence signal resulting from the CPK3–bHLH107 interaction (Figure 3A). Moreover, a competitive pull‐down assay revealed that the amount of MBP‐tagged bHLH107 proteins recovered by a constant quantity of HIS‐CPK3 progressively decreased with increasing amounts of GST‐tagged GRF1 protein (Figure 3B). These results suggest that GRF1 impairs the interaction between bHLH107 and CPK3.

**Figure 3:**
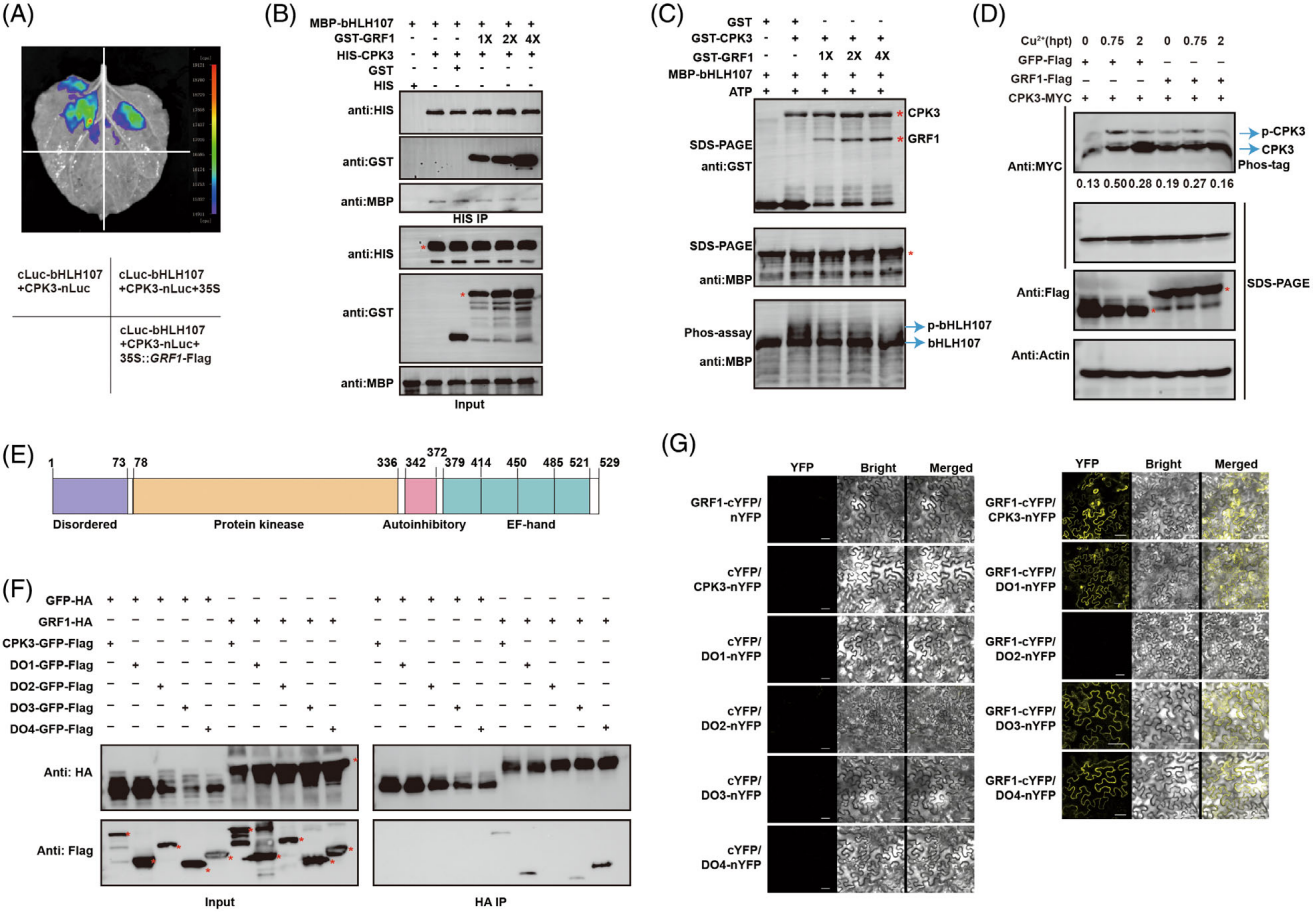
GRF1 impaired CPK3 kinase activity. (A, B) Luciferase complementation (A) and pull‐down (B) assays showing that GRF1 inhibits the interaction between bHLH107 and CPK3. Coexpression of cLuc‐bHLH107 and CPK3‐nLuc with either 35S::*GRF1*‐*Flag* or an empty vector of *35S::Flag* (PL43_PS1300‐Flag) as a control. For the pull‐down assay, MBP‐tagged bHLH107 was incubated with a constant quantity of HIS‐tagged CPK3 under the condition of increasing the quantity of GST‐tagged GRF1, after which the precipitated fractions were analyzed by immunoblotting with anti‐GST, anti‐MBP, and anti‐HIS antibodies. (C, D) *In vitro* (C) and *in vivo* (D) kinase activity assays combined with Phos‐tag gel immunoblotting assays showing that GRF1 inhibits CPK3 kinase activity. bHLH107 phosphorylation was inhibited by supplementation with increasing GST‐tagged GRF1 protein. Recombinant GST‐CPK3 and MBP‐bHLH107 proteins and increased amounts of GST‐GRF1 were incubated in kinase buffer containing ATP (C). For the *in vivo* assay, CPK3‐MYC was transiently coexpressed with GRF1‐Flag in *N. benthamiana*, and CPK3‐MYC was coexpressed with GFP‐Flag as a control (D). Phosphorylated p‐bHLH107 and p‐CPK3 were analyzed in a Phos‐tag gel, and are marked with blue arrows. (E) Illustration of the CPK3 protein domain. DO1 represents the disordered domain, DO2 represents the protein kinase domain, DO3 represents the autoinhibitory domain, and DO4 represents the EF‐hand domain. (F, G) Interactions between GRF1 and full‐length CPK3, DO1, DO2, DO3, and DO4 were determined by co‐IP (F) and BiFC (G) assays. The methods used were previously described in Figure 2.

Cu^2+^ treatment activated CPK3 phosphorylation (Figure 2E), which in turn phosphorylates GRF1 and bHLH107 (Swatek et al., 2014; Yu et al., 2026), and impaired the CPK3–GRF1 interaction (Figure 2C). Here, we found that GRF1 also repressed the CPK3–bHLH107 interaction; therefore, we hypothesized that GRF1 represses CPK3 enzymatic activity under normal conditions, and that Cu^2+^ treatment alleviates this repression. To test this hypothesis, we performed an *in vitro* kinase assay using GST‐tagged CPK3, MBP‐tagged bHLH107, and increasing amounts of GST‐tagged GRF1. We observed that CPK3 phosphorylated bHLH107, whereas the level of phosphorylated bHLH107 decreased with increasing amounts of GST‐GRF1 (Figure 3C), indicating that GRF1 impaired CPK3 kinase activity. Furthermore, we transiently coexpressed CPK3‐MYC with either GRF1‐Flag or GFP‐Flag and found that GRF1‐Flag, but not GFP‐Flag, decreased the level of phosphorylated CPK3 after Cu^2+^ treatment (Figure 3D). Overall, these results suggest that GRF1 suppresses the CPK3–bHLH107 interaction and impairs CPK3 kinase activity.

CPK3 contains four domains: disordered domain (DO1), protein kinase domain (DO2), autoinhibitory domain (DO3), and EF‐hand domain (DO4) (Figure 3E). To identify the domain(s) responsible for the interaction with GRF1, we performed co‐IP assays. As shown in Figure 3F, HA‐tagged GRF1 was precipitated by Flag‐tagged full‐length CPK3, DO1, DO3, and DO4, but not by DO2. These interactions were further confirmed by BiFC assays in *N. benthamiana* (Figure 3G). Collectively, our results suggest that GRF1 may bind to the DO1, DO3, and DO4 domains of CPK3, thereby impairing its kinase activity.

### Phosphorylated bHLH107 dissociates from GRF1

A previous study demonstrated that Cu^2+^ activates the phosphorylation of bHLH107 at the Ser‐62 and Ser‐72 residues (Yu et al., 2026). However, whether phosphorylated bHLH107 loses its interaction with GRF1 and its phospho‐mutants remains elusive. To address this, we performed a series of BiFC assays and observed that GRF1, GRF1‐4A, and GRF1‐4D interacted with wild‐type bHLH107, the double phospho‐defective mutant protein bHLH107^S62/72A^ (bHLH107‐2A) and the double phosphomimetic mutant protein bHLH107^S62/72D^ (bHLH107‐2D) (Figure 4A). These interactions were further validated by two independent co‐IP assays in *N. benthamiana* (Figure 4B, Figure S3). However, *in vitro* pull‐down assays, which assess direct protein–protein interactions, revealed that GRF1 and its phospho‐mutants directly interacted with bHLH107 and bHLH107‐2A, but failed to bind bHLH107‐2D (Figure 4C). In addition, to determine whether the two CPK3 recognition motifs in bHLH107 (RINS and KLLS) serve as key binding sites for GRF1, we deleted these motifs and performed LCA and pull‐down assays. The results indicated that deletion of these motifs did not affect the interaction between GRF1 and bHLH107 (Figure S4A,B).

**Figure 4:**
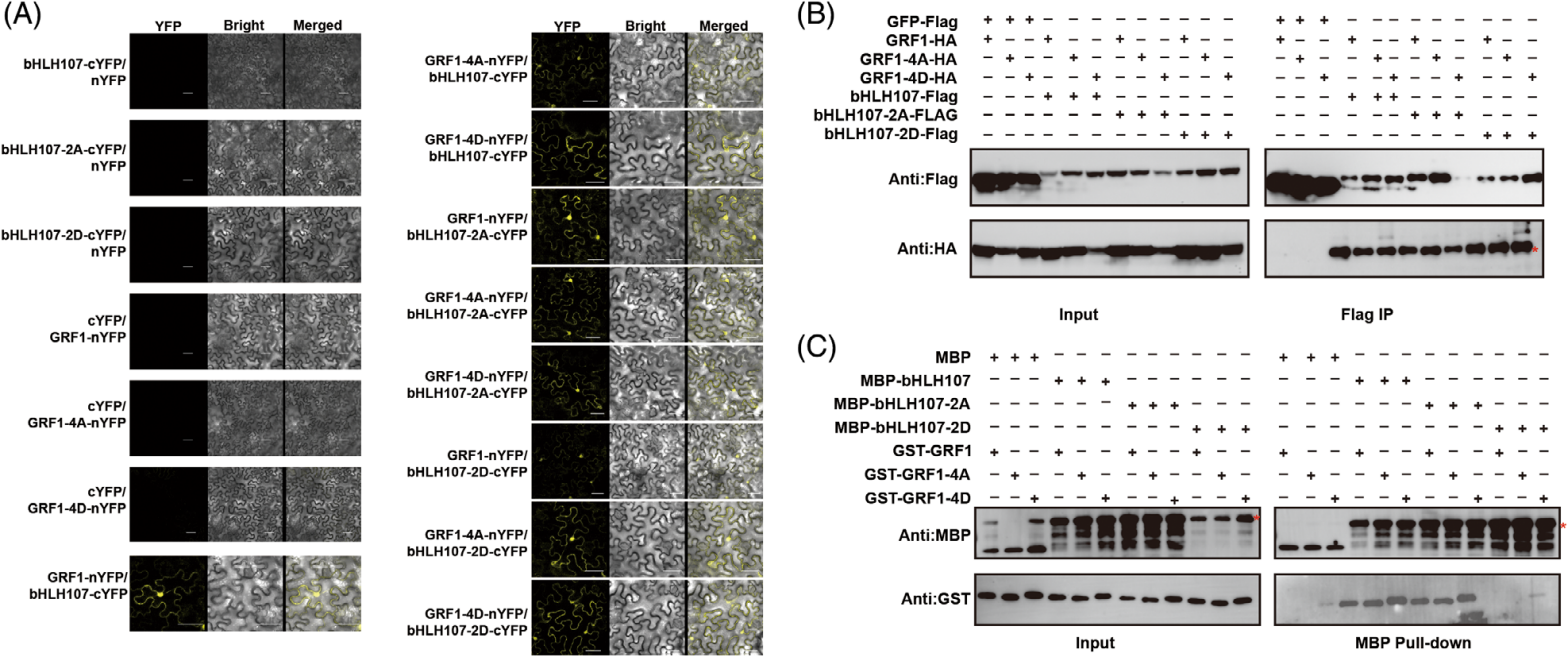
Phosphorylated bHLH107 dissociates from GRF1. (A–C) Interactions between bHLH107, bHLH107‐2A, bHLH107‐2D with GRF1, GRF1‐4A, GRF1‐4D were performed with BiFC (A), co‐IP (B), and pull‐down (C) assays. The methods used are as previously described in Figure 2.

### Cu^2+^ and activated CPK3 mediate disruption of the GRF1‐bHLH107 complex

Previous studies have demonstrated that bHLH107‐2D exhibits enhanced transcriptional activity capacity toward *ACS8* expression (Yu et al., 2026). Consistent with our finding that phosphorylated bHLH107 lost the ability to directly interact with GRF1 (Figure 4C), we next examined the effect of GRF1 on the transcriptional activation capacity of bHLH107. Relative luciferase activity assays revealed that GRF1 impaired the bHLH107‐mediated transcription level of CuRE (the luciferase reporter driven by *CuRE:35S mini*) (Figure 5A,B). Furthermore, Electrophoretic Mobility Shift Assay (EMSA) analysis demonstrated that GRF1 inhibited the DNA‐ binding ability of bHLH107, as evidenced by reduced binding to the CuRE *cis*‐element sequence (Figure 5C). We therefore speculated that Cu^2+^‐activated CPK3 may disrupt the GRF1‐bHLH107 complex. To test this, we performed semi*‐in vivo* competitive pull‐down assays combined with Phos‐tag gel analysis (Figure 5D). Prokaryotically expressed MBP‐tagged bHLH107 and GST‐tagged GRF1, together with *N. benthamiana* leaves expressing *35S::CPK3‐MYC*, were harvested. Phosphorylated and unphosphorylated CPK3‐MYC proteins were obtained from plants treating with or without 100 μM Cu^2+^, respectively. The purified CPK3‐MYC proteins were then incubated with GST‐GRF1 and MBP‐bHLH107 at 4°C for 1 h, after which MBP‐bHLH107 was pulled down using anti‐GST beads. Quantification of the captured MBP‐bHLH107 protein revealed that Cu^2+^‐activated CPK3‐MYC competed more effectively for bHLH107 binding than unphosphorylated CPK3‐MYC or the MYC control (Figure 5D). These findings were further corroborated by *in vitro* competitive pull‐down assays using ATP‐autoactivated CPK3 (Figure 5E). Collectively, these results indicate that Cu^2+^‐activated CPK3 phosphorylation impaired the GRF1–bHLH107 interaction.

**Figure 5:**
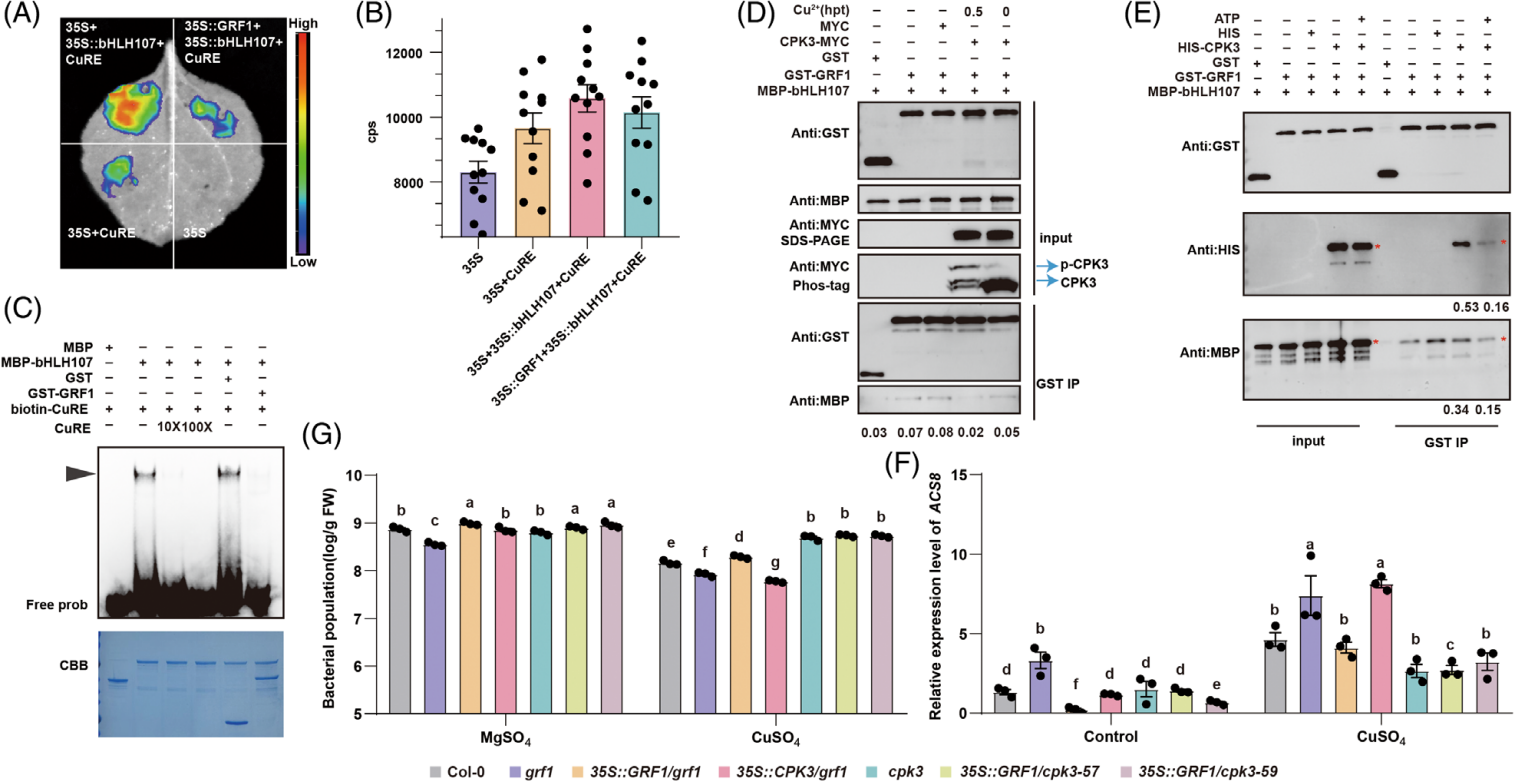
Cu^2+^ and activated CPK3 mediate disruption of the GRF1‐bHLH107 complex. (A, B) Representative image (A) and statistical data (B) of relative luciferase activity assay showing that GRF1 inhibits the transcriptional activation capacity of bHLH107 in regulating *ACS8*. Coexpression of bHLH107‐HA and *CuRE:35S mini‐Luc* with either 35S::*GRF1*‐*Flag* or the empty vector of *35S::Flag* (PL43_PS1300‐Flag) as a control (means ± SEMs; *n* = 11). (C) EMSA showing that GRF1 inhibited the DNA binding ability of bHLH107. The CuRE sequence (5′‐CCAAAAGAAGAAGAAAAACCAAAAGAAGAAGAAAAA‐3′) was designed and labeled with biotin. (D, E) *Semi‐in vivo* (D) and *in vitro* (E) pull‐down assays showing that phosphorylated CPK3 disrupts the interaction between GRF1 and bHLH107. Prokaryotically expressed HIS‐CPK3 was incubated in kinase buffer containing ATP, with HIS was used as a control. Transiently expressed CPK3‐MYC in *N. benthamiana* was activated by treatment with 100 μM CuSO_4_, and MYC expression was used as a control. Phosphorylated CPK3 was analyzed with a Phos‐tag gel and is marked with a blue arrow. (F, G) Relative *ACS8* expression (F) and bacterial load of *Pst* DC3000 (G) in wild‐type Col‐0, the *grf1* mutant, *35S::GRF1/grf1*, *35S::CPK3/grf1*, *cpk3*, and *35S::GRF1/cpk3*. The methods used were previously described in Figure 1.

To further establish the genetic roles of CPK3 and GRF1 in Cu^2+^‐mediate signaling, we generated *35S::GRF1/cpk3* and *35S::CPK3/grf1* lines by introducing the *35S::GRF1* construct into the *cpk3* mutant and the *35S::CPK3* construct into the *grf1* mutant, respectively (Figure S2B). We first examined the relative transcript levels of *ACS8* in Col‐0, *grf1*, *35S::GRF1/grf1*, *35S::CPK3/grf1*, *cpk3*, and *35S::GRF1/cpk3* following CuSO_4_ treatment. The expression level of *ACS8* in *35S::CPK3/grf1* was higher than that in Col‐0 (Figure 5F), and accordingly, *35S::CPK3/grf1* exhibited enhanced resistance to *Pst* DC3000 compared with Col‐0 (Figure 5G). These results further support that loss of *GRF1* combined with overexpressing *CPK3* activates the Cu^2+^‐induced *ACS8* transcription and enhances Cu^2+^‐mediated resistance to *Pst* DC3000. In contrast, compared with *35S::GRF1/grf1* and Col‐0, *35S::GRF1/cpk3* showed slightly reduced Cu^2+^‐induced *ACS8* expression and a modest increase in *Pst* DC3000 bacterial growth (Figure 5F,G). These findings suggest that loss of *CPK3* combined with overexpressing *GRF1* repressed Cu^2+^‐induced *ACS8* transcription and resistance to *Pst* DC3000. Collectively, our results demonstrate that Cu^2+^ and activated CPK3 promote the disruption of the GRF1‐bHLH107 complex.

### bHLH107 and its phospho‐mutants interact with CPK3

We previously demonstrated that CPK3 interacts with and phosphorylates bHLH107 at serine residues 62 and 72 (Yu et al., 2026). To further characterize the interaction between CPK3 and bHLH107 and its phospho‐mutants, we performed BiFC assays and observed that bHLH107, bHLH107‐2A, and bHLH107‐2D all interact with CPK3 (Figure 6A). These interactions were further validated by co‐IP and LCA assays in *N. benthamiana* (Figure 6B–D). To determine whether the phosphorylation status of CPK3 affects its interaction with bHLH107, we performed *in vitro* pull‐down assays using autophosphorylated and unphosphorylated CPK3. The results revealed that both phosphorylated and unphosphorylated CPK3 directly interacted with bHLH107, bHLH107‐2A, and bHLH107‐2D (Figure 6E). In summary, these results indicate that CPK3 interacted with bHLH107 regardless of the phosphorylation status of either protein.

**Figure 6:**
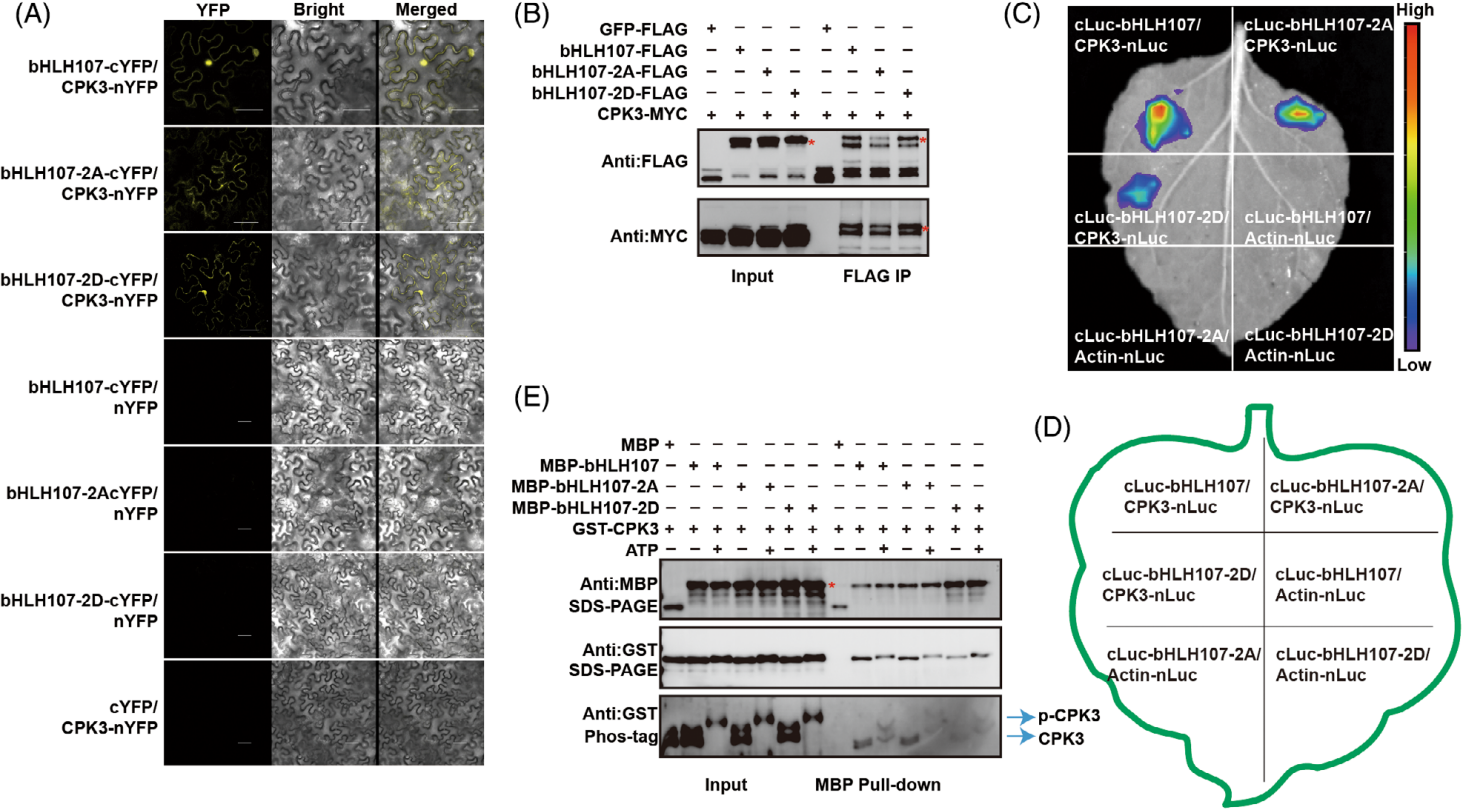
bHLH107 and its phospho‐mutants interact with CPK3. (A–E) Interactions of bHLH107, bHLH107‐2A, and bHLH107‐2D with CPK3 were evaluated by BiFC (A), co‐IP (B), LCA (C‐D), and pull‐down (E) assays. The methods used were previously described.

## DISCUSSION

Copper ions are well‐established trace elements that participate in plant photosynthesis (Yruela, 2013). We previously demonstrated that CPK3 phosphorylates bHLH107, thereby increasing *ACS8* transcription to positively regulate CuTI (Yu et al., 2026). Moreover, bHLH107‐mediated CuTI is genetically located downstream of copper transporters, such as the redundant COPT4‐COPT1 and COPT4‐COPT3 complexes (Xia et al., 2026). However, the mechanisms underlying the fine‐tuning of the CPK3‐mediated phosphorylation of bHLH107 have remained unclear. In this study, we demonstrated that the Arabidopsis 14‐3‐3 protein GRF1 participates in CuTI (Figure 1). Under normal conditions, GRF1 interacts with both CPK3 and bHLH107 (Figures 1 and 2), attenuating CPK3 kinase activity and bHLH107 transcriptional activity (Figures 3 and 5), thereby maintaining the cytosolic complex in an ‘OFF’ state. Upon Cu^2+^ perception, the signaling module transitions to an ‘ON’ state, accompanied by CPK3 phosphorylation (Figure 2). Subsequently, phosphorylated CPK3 competes with GRF1 for bHLH107 binding and phosphorylates bHLH107 at Ser‐62 and Ser‐72, promoting dissociation of the GRF1‐bHLH107 complex, enhancing the transcriptional activity of bHLH107 (Figures 4, 5, 6), upregulating the *ACS8* transcription, and activating CuTI in Arabidopsis. Collectively, these findings support a model in which the GRF1‐CPK3‐bHLH107 module governs Cu^2+^‐induced defense responses in Arabidopsis (Figure 7).

**Figure 7:**
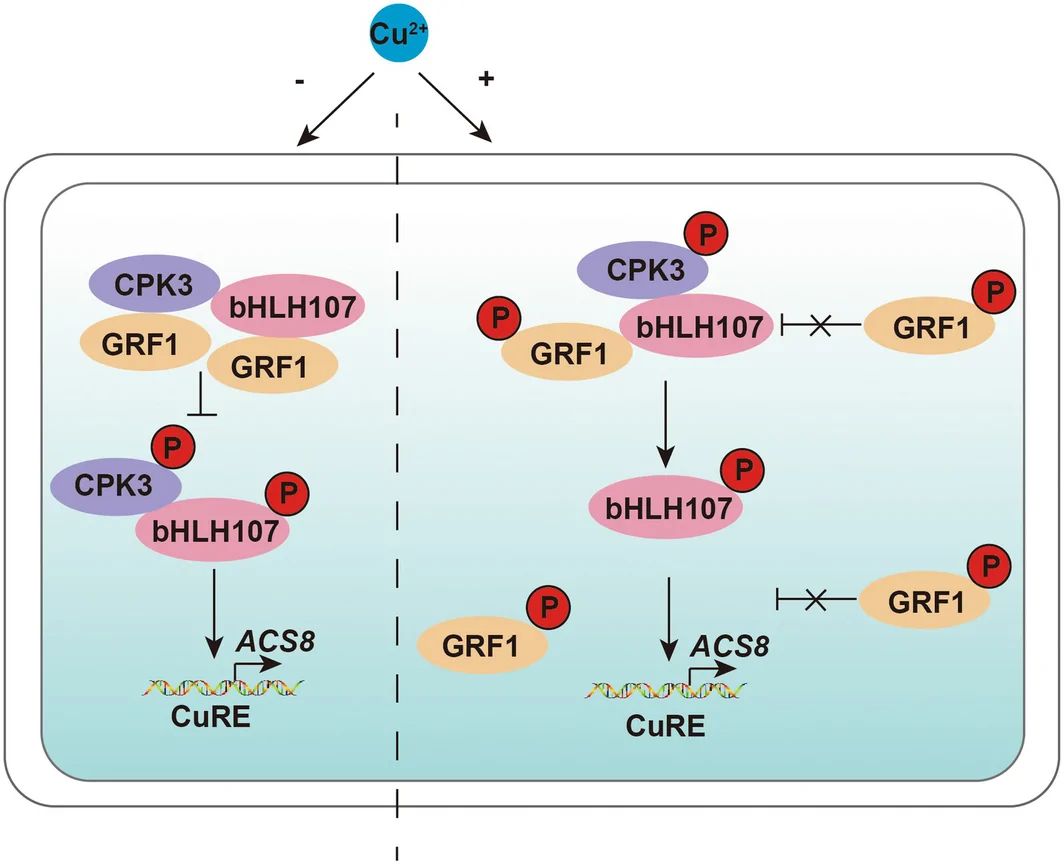
A GRF1‐mediated interaction network for CPK3‐bHLH107 in CuTI. Under normal conditions (without Cu^2+^ treatment or ‘OFF’ status), GRF1 interacts with both CPK3 and bHLH107, inhibiting CPK3 kinase activity and bHLH107 transcriptional activity, respectively. Upon Cu^2+^ treatment, CPK3 is phosphorylated and subsequently phosphorylates bHLH107 at Ser62 and Ser72, leading to the dissociation of bHLH107 from GRF1, promoting the upregulation of the *ACS8* transcription and triggering a series of downstream immune responses in Arabidopsis.

CPK3 is a Ca^2+^‐sensor that regulates multiple intracellular signaling pathways, such as those involved in stomatal movement, salt and cold stress (Li, Wang, et al., 2025; Mehlmer et al., 2010; Ming et al., 2025). Previous studies have shown that salt and cold stress signals activate CPK3 kinase activity in a Ca^2+^‐dependent manner in Arabidopsis (Mehlmer et al., 2010; Ming et al., 2025), and that CPK3 in turn positively regulates Ca^2+^ signaling by directly phosphorylating the Ca^2+^ channels CYCLIC‐NUCLEOTIDE‐GATED CHANNEL 5 (CNGC5) and CNGC6 (Ming et al., 2025). As a stimulus similar to PAMP, CuTI is partially dependent on CPK3 and involves Ca^2+^ signaling (Liu et al., 2015; Yu et al., 2026). Cu^2+^ activates CPK3 phosphorylation within 0.5 h (Figure 2E). Notably, pretreatment with 10 mM LaCl_3_ decreases but did not completely abolish Cu^2+^‐induced CPK3 phosphorylation at 0.5 h, and the phosphorylation level subsequently peaked at 1 h (Figure 2E). This findings indicate that blocking calcium channels delays, rather than completely inhibits, Cu^2+^‐ stimulated CPK3 phosphorylation. This mechanism is distinct from that underlying salt/cold stress‐induced CPK3 activation (Mehlmer et al., 2010; Ming et al., 2025). In addition, CPK3 is localized in guard cells and plays a key role in stomatal movement (Li, Wang, et al., 2025). CPK3 also regulates actin cytoskeleton organization by phosphorylating actin‐depolymerization factor 4 (ADF4) and participates in both pattern‐triggered immunity (PTI) and effector‐triggered immunity (ETI), being required for flg22‐induced stomatal closure and resistance to *Pst* DC3000 carrying the effector AvrPphB (Lu et al., 2020). Arabidopsis CPK3 is crucial for both ABA‐induced stomatal closure (Zhang et al., 2024) and ethylene‐induced stomatal closure (Li, Wang, et al., 2025). Upstream of ethylene‐induced stomatal closure, CPK3 interacts with RBOHF to regulate the hydrogen peroxide (H_2_O_2_)‐nitric oxide (NO) signaling module (Li, Wang, et al., 2025). In our previous studies, we demonstrated that the CPK3‐bHLH107 module upregulates *ACS8* transcription, leading to pronounced *ACS8* expression in guard cells, which enhances ethylene signaling to suppress ABA synthesis and promote stomatal reopening (Zhang et al., 2018; Liu et al., 2020; Yu et al., 2026). Coincidentally, Cu^2+^ can also rapidly induce a ROS burst (Liu et al., 2015). Thus, the CPK3‐RBOHF‐H_2_O_2_‐NO module that mediates ethylene‐induced stomatal closure is quite different from the Cu^2+^‐activated CPK3‐bHLH107 module that promotes stomatal reopening.

As spider in a web of phosphorylation, 14‐3‐3 s coordinate multiple phosphorylation events to participate in plant growth and stress responses (de Boer et al., 2013). Accumulating evidence indicates that 14‐3‐3 proteins can regulate enzyme activity: For instance, the cytosolic invertase CINV1 is phosphorylated by CPK3/21 in response to light, which increases its binding ability for 14‐3‐3 s; this interaction, in turn, potentiates CINV1 enzymatic activity (Gao et al., 2014). Some 14‐3‐3 s inhibit nitrate reductase (NR) activity (Lambeck et al., 2010). However, the phosphomimetic variant 14‐3‐3χ‐S72D fails to repress the activity of phosphorylated NR (Swatek et al., 2014). In addition, Arabidopsis 14‐3‐3λ and 14‐3‐3κ interact with SOS2 and suppress its kinase activity. Under salt stress, the interaction between salt overly sensitive (SOS) 2 and 14‐3‐3 proteins is weakened, thereby promoting SOS2 kinase activation (Zhou et al., 2014). SOS2‐LIKE PROTEIN KINASE 5 (PKS5) interacts with and phosphorylates SOS2 at Ser294, enhances the association between SOS2 and 14‐3‐3 s, and thereby repressing SOS2 activity. Under salt stress, 14‐3‐3 s bind more strongly to PKS5, inhibiting PKS5 kinase activity and ultimately alleviating PKS5‐mediated repression of SOS2 (Yang et al., 2019). The N‐terminus and C‐terminus regions of MAPKKK5 undergo intramolecular interaction that blocks access of receptor‐like cytoplasmic kinases (RLCKs) to the MAPKKK5 C‐terminal region. 14‐3‐3 proteins alleviate this autoinhibition, facilitating the interaction between RLCKs and the MAPKKK5 C‐terminus, thereby promoting MAP kinase activation and enhancing plant disease resistance (Dong et al., 2023). On the other hand, 14‐3‐3 s also modulate the stability and translocation of CDPKs. PHS induces CPK3 activation, and phosphorylation of the conserved serine residue corresponding to Ser58 of GF14ζ disrupts the 14‐3‐3 s‐CPK3 complex, triggering CPK3 dissociation from 14‐3‐3 s and leading to CPK3 degradation (Lachaud et al., 2013). CPK12 is rapidly activated during hypoxia, shuttling from the cytoplasm to the nucleus, a process negatively regulated by 14‐3‐3κ (Fan et al., 2023). In this study, we found that Cu^2+^ induces CPK3 phosphorylation, and its dissociation from GRF1 (Figure 2). However, we did not observe the degradation of CPK3 (Figure 2D); instead, we detected increased phosphorylation of bHLH107, another CPK3 substrate (Yu et al., 2026). Interestingly, in the ‘OFF’ status (without Cu^2+^ treatment), GRF1 interacts with CPK3 to inhibit its kinase activity (Figure 3). This reflects an important role for GRF1 in fine‐tuning CPK3 activity to prevent unexpected immune activation in plants.

In addition to directly regulating kinase function, 14‐3‐3 s also regulate the subcellular location of transcription factors through protein–protein interactions and phosphorylation events. BIN2‐mediated phosphorylation of BZR1/BES1 (BZR2) promotes their binding to 14‐3‐3 proteins. Mutation of the BIN2 phosphorylation site in BZR1 abolishes 14‐3‐3 association and consequently enhances the nuclear accumulation of BZR1 (Gampala et al., 2007). The bZIP transcription factor RSG interacts with 14‐3‐3 proteins. The RSG mutant is unable to bind 14‐3‐3 and predominantly localizes in the nucleus and exhibits higher transcriptional activity than the wild‐type (Igarashi et al., 2001). In soybean, endogenous 14‐3‐3 proteins physically interact with GmMYB176 and modulate its subcellular localization, thereby regulating isoflavonoid biosynthesis (Li et al., 2012). In our study, we found that GRF1 inhibits CPK3 kinase activity. Besides, GRF1 directly interacts with unphosphorylated bHLH107, and this interaction both directly and indirectly suppresses the transcriptional activity of bHLH107 and its binding to the CuRE *cis*‐element on the *ACS8* promoter (Figures 4 and 5). In contrast, Cu^2+^ treatment triggers CPK3‐mediated bHLH107 phosphorylation, resulting in its dissociation from GRF1 and activation of the transcription factor, thereby lighting the immune response.

As reported in our previous work (Yu et al., 2026), HY5 facilitates bHLH107‐mediated activation of *ACS8* expression by binding to the ACE‐box, which is adjacent to the CuRE element. In this study, transcriptional activation assays revealed that HY5 did not enhance bHLH107‐mediated CuRE activation (Figure S5). These results indicate that HY5 specifically binds to the ACE‐box to assist bHLH107 in regulating *ACS8* expression. Moreover, coexpressing HY5 and GRF1 resulted in reduced bHLH107‐activated CuRE expression (Figure S5), implying that GRF1 may directly or indirectly interact with bHLH107 in the nucleus. These findings further suggest that GRF1‐mediated repression of bHLH107 transcriptional activity toward CuRE elements may be independent of HY5 regulation.

In summary, building upon the previously identified CPK3‐bHLH107 module that confers CuTI, we further revealed that the 14‐3‐3 protein GRF1 manipulates the ‘ON’ or ‘OFF’ status of this module in a phosphorylation‐dependent manner.

## MATERIALS AND METHODS

### Plant materials and bacterial growth assays

The wild‐type Arabidopsis used in this study was Columbia (Col‐0). The *grf1* mutant (SALK_018664C) was obtained from the Arabidopsis Biological Resource Center (TAIR‐ABRC). The primers used for PCR‐based positive identification of the mutant plants are listed in Table S1. Planting conditions and methods were carried out as previously described (Zhang et al., 2018). Briefly, Arabidopsis seeds were surface‐sterilized and sown on half‐strength Murashige and Skoog (1/2 MS) medium, then incubated under a 16 h light/8 h dark photoperiod (20°C–22°C). After 10 days, seedlings were transplanted into soil for further development.

As previously described (Yu et al., 2026), *Pst* DC3000 was inoculated into the leaves of 4‐week‐old Arabidopsis plants using a needleless syringe with a bacterial suspension containing 10^6^ CFU ml^−1^. Leaf samples were collected at 0 and 3 days post‐inoculation (dpi) for bacterial assays and colony counting.

### Plasmid construction and plant transformation

To generate the 35S::*GRF1‐Flag* construct, the *GRF1* coding sequence (CDS) was amplified from Arabidopsis complementary DNA (cDNA) and inserted into the PS1300T‐bFlag vector (*Eco*R I‐*Sac* I) as previously reported (Yu et al., 2026). Site‐directed mutagenesis of 35S::*GRF1‐*4A‐*Flag* and 35S::*GRF1‐*4D‐*Flag* was performed using the Mut Express II Fast Mutagenesis Kit V2 (Vazyme, Nanjing, China). For the *ProGRF1::gGRF1‐Flag* construct, the full‐length genomic *GRF1* sequence was amplified from Arabidopsis genomic DNA and inserted into the PC1300TB‐bFlag vector (*Eco*R I‐*Sac* I). All primers used for construct generation are listed in Table S1.

To generate the transgenic plants, the 35S::*GRF1‐Flag*, 35S::*GRF1‐*4A‐*Flag*, 35S::*GRF1‐*4D‐*Flag*, and *ProGRF1::gGRF1‐Flag* constructs were individually transformed into *Agrobacterium tumefaciens* strain GV3101 (Coolaber, Beijing, China). The transformed *Agrobacterium* cultures were grown at 28°C, and the bacterial suspensions were adjusted to an OD_600_ of 0.6–0.8 using infection solution (50 g/L sucrose, 0.02% Silwet L‐77). The *grf1* inflorescences were subsequently submerged in the infection solution for 20 s and incubated in the dark overnight.

### RNA extraction and RT‐qPCR

Total RNA was extracted from 4‐week‐old Arabidopsis plants using a Plant RNA Kit (OMEGA Biotek, Norcross, USA). cDNA was synthesized using the ABScript Neo RT Master Mix for qPCR with gDNA Remover (ABclonal, Wuhan, China). The relative expression levels of target genes were determined using 2X Universal SYBR Green Fast qPCR Mix (ABclonal) on a CFX Connect™ Real‐Time System and a CFX384™ Real‐Time System (Bio‐Rad Laboratories, Hercules, USA). The primers used for RT‐qPCR are listed in Table S1.

### BiFC assays

The pairs of GRF1‐YFP^N^, GRF1‐YFP^C^, GRF1‐4A‐YFP^N^, GRF1‐4A‐YFP^C^, GRF1‐4D‐YFP^N^, GRF1‐4D‐YFP^C^, bHLH107‐YFP^N^, bHLH107‐YFP^C^, bHLH107‐2A‐YFP^N^, bHLH107‐2A‐YFP^C^, bHLH107‐2D‐YFP^N^, bHLH107‐2D‐YFP^C^, CPK3‐YFP^N^, YFP^N^, and YFP^C^ were coexpressed in *N. benthamiana* leaves for 48 h. YFP fluorescence (514 nm) was detected using a Microsystems confocal microscope SP8 (Leica, Wetzlar, Germany) equipped with a 40× objective.

### Luciferase complementation assays

To generate the *bHLH107‐cLuc* construct, the bHLH107 CDS was amplified from Arabidopsis cDNA and inserted into the pCAMBIA1300‐cLuc vector (*Kpn*I‐*Bam*HI). To generate the *nLuc‐CPK3* construct, the *CPK3* CDS was amplified from Arabidopsis cDNA and inserted into a pCAMBIA1300‐nLuc vector (*Sac*I‐*Xba*I) as previously described (Yu et al., 2026).

GV3101 carrying cLuc‐bHLH107 and CPK3‐nLuc was coexpressed in *N. benthamiana* for 48–60 h. A luciferase substrate solution (100 mM Tris HCl, pH 7.8; 150 μM ATP; 10 mM MgCl_2_; 3 g/L D‐luciferin potassium salt) was sprayed onto the back of *N. benthamiana*, and fluorescence was detected using a NightSHADE LB985 plant living molecular imaging system (Berthold, Germany).

### Pull‐down assays

The GST‐CPK3, GST‐GRF1, MBP‐GRF1, MBP‐GRF1‐4A, MBP‐GRF1‐4D, MBP‐bHLH107, MBP‐bHLH107‐2A, and MBP‐bHLH107‐2D constructs were prokaryotically expressed and purified from *E. coli* BL21 (DE3). For pull‐down assay, MBP‐tagged proteins were incubated with interacting proteins at 4°C for 1 h, followed by incubation with MBP‐agarose beads (Beijing BioRab Technology, China) at 4°C for an additional 1.5 h. Subsequently, the beads were washed with PBS (pH 7.4). The eluted proteins were detected by immunoblotting with anti‐GST (Proteintech, Wuhan, China) and anti‐MBP (Proteintech) antibodies.

### Co‐IP assays

The pairs of GRF1‐Flag, GRF1‐4A‐Flag, GRF1‐4D‐Flag, bHLH107‐HA, bHLH107‐2A‐HA, bHLH107‐2D‐HA, CPK3‐MYC, GFP‐Flag, and GFP‐HA were coexpressed in *N*. *benthamiana* leaves for 48 h. Total protein was extracted with IP buffer (50 mM Tris HCl (pH 7.5), 150 mM NaCl, 5 mM MgCl_2_, 5% glycerol, 0.1% NP‐40, 1 mM DTT, 1 mM PMSF, and 1× protein inhibitor cocktail). Protein extracts were incubated with protein A/G magnetic beads (MedChemExpress, Shanghai, China) that had been preincubated with anti‐MYC, anti‐FLAG, or anti‐HA antibodies (Proteintech) for 2 h at 4°C. The beads were subsequently washed eight times with IP buffer. The eluted samples were detected by immunoblotting with anti‐HA (Proteintech), anti‐FLAG (Vazyme), and anti‐MYC (Proteintech) antibodies.

### *In vitro* kinase assay

*In vitro* kinase assay was performed as previously described (Yu et al., 2026). Briefly, approximately 1 μg of GST‐CPK3 protein was incubated in kinase assay buffer (50 mM Tris HCl (pH 7.5), 1 mM CaCl_2_, 10 mM MgCl_2_, and 1 mM DTT) with or without 250 μM ATP at 30°C for 1 h. The reactions were stopped by adding 25 μL 5x SDS loading buffer and boiling at 95°C for 10 min. The reaction products were subsequently used for pull‐down assays. After that, the proteins were separated on 8% SDS‐PAGE gels and 6% Phos‐tag gels (Vazyme) and detected by immunoblotting with anti‐MBP and anti‐GST antibodies (Proteintech), respectively.

### DNA–protein interaction assays

For luciferase assays, 35S::*bHLH107‐HA*, 35S::*GRF1‐FLAG*, and *CuRE‐35S mini:Luc* were coexpressed in *N. benthamiana* for 48 h. Luminescence was detected using a NightSHADE LB985 plant living molecular imaging system. Electrophoretic mobility shift assay (EMSA) was performed according to previously reported methods (Yu et al., 2026). The biotin‐labeled probes were synthesized by Sangon Biotech (China) and are listed in Table S1.

### Statistical analysis

Unless otherwise specified, all experiments were performed in triplicates. Data are presented as mean ± SEM. Student's *t*‐test was used for two‐group comparisons, and one‐way or two‐way ANOVA was applied for multi‐group analyses.

## AUTHOR CONTRIBUTIONS

YY and ZC designed the experiments. YY, HX, and RD performed the experiments. YY and HL performed the data analysis. YY, ZC, and HC wrote the manuscript. All authors read and approved the final manuscript.

## CONFLICT OF INTEREST

The authors declare no conflict of interest.

## Supporting information


**Figure S1.** Screening of 14‐3‐3 proteins interacting with bHLH107. (A) List of 14‐3‐3 proteins identified by LC–MS/MS as bHLH107 interactors, corresponding to Figure 1. (B) Pull‐down assay showing that GRF1 directly interacts with bHLH107 *in vitro*, MBP protein was used as the bait protein for the negative control. (C) Relative *ACS8* expression in wild‐type Col‐0 and *grf5*. Samples was harvested at 2 h after treated with or without CuSO_4_. Expression levels were normalized to *Actin2* (*At3g18780*). Error bars represent the means ± SEMs (*n* = 3). Asterisks indicate significant differences compared with the control of Col‐0 (two‐way ANOVA). (D‐E) LCA assay analyzing the interaction strength between GRF1 and bHLH107.
**Figure S2.** Characterization of transgenic lines at RNA and protein levels. (A, B) Molecular identification of transgenic lines at the RNA and protein levels. Error bars represent the means ± SEMs (*n* ≥ 2). Asterisks indicate significant differences compared with Col‐0 (one‐way ANOVA). Ponceau S staining was used to verify equal protein loading. (C) Relative expression level of GRF1 in Col‐0. Samples were collected after treatment with 10 μM and 100 μM CuSO_4_ for 0, 0.5, 1, and 2 h. Error bars represent the means ± SEMs (*n* = 3). Asterisks indicate significant differences compared with the control of Col‐0 (one‐way ANOVA).
**Figure S3.** Co‐IP assays showing that bHLH107, bHLH107‐2A and bHLH107‐2D interact with GRF1, GRF1‐4A and GRF1‐4D.
**Figure S4.** Deletion of RINS and KLLS motifs in bHLH107 does not affect its interaction with GRF1. (A, B) pull‐down (A) and LCA (B) assays showing that deletion of the RINS and KLLS motifs of bHLH107 (bHLH107‐8mute) does not affect its interaction with GRF1. The methods used were previously described.
**Figure S5.** GRF1‐mediated inhibition of bHLH107 transactivation activity is independent of HY5. Relative luciferase activity of *CuRE‐35S mini:Luc*, means ± SEMs; *n* = 12.
**Table S1.** Primers used in this study.

## Data Availability

All relevant data can be found within the manuscript and its Supporting Materials.
