# Supplementary material for: GRF1 fine‐tunes the copper‐responsive transcription module of CPK3‐bHLH107 by manipulating interaction and phosphorylation
Source: Plant J. 2026 Sep 30;127(6):e71150. doi: 10.1111/tpj.71150 (PMC13627851; doi:10.1111/tpj.71150)
Supplement: Supplementary file 1 [file TPJ-127-0-s001.pdf]

GRF1 fine-tunes the copper-responsive transcription module of CPK3-bHLH107 by manipulating interaction and phosphorylation

Yue Yu[1,2], Haoran Xia[1,2], Ran Dong[3], Haifeng Liu[4], Xiangsong Chen[1,2], Zhaohui Chu[1,2]*

[1]State Key Laboratory of Hybrid Rice, Hubei Provincial Research Center for Basic Biological Sciences, College of Life Sciences, Wuhan University, Wuhan 430072, China

[2]Hubei Hongshan Laboratory, Wuhan 430070, China

[3]Shandong Institute of Pomology, Tai'an, 271000, China

[4]College of Agronomy, Shandong Agricultural University, Taian 271018, China

Correspondence:

Zhaohui Chu, zchu77@whu.edu.cn

ORCID ID:0000-0001-8320-7872

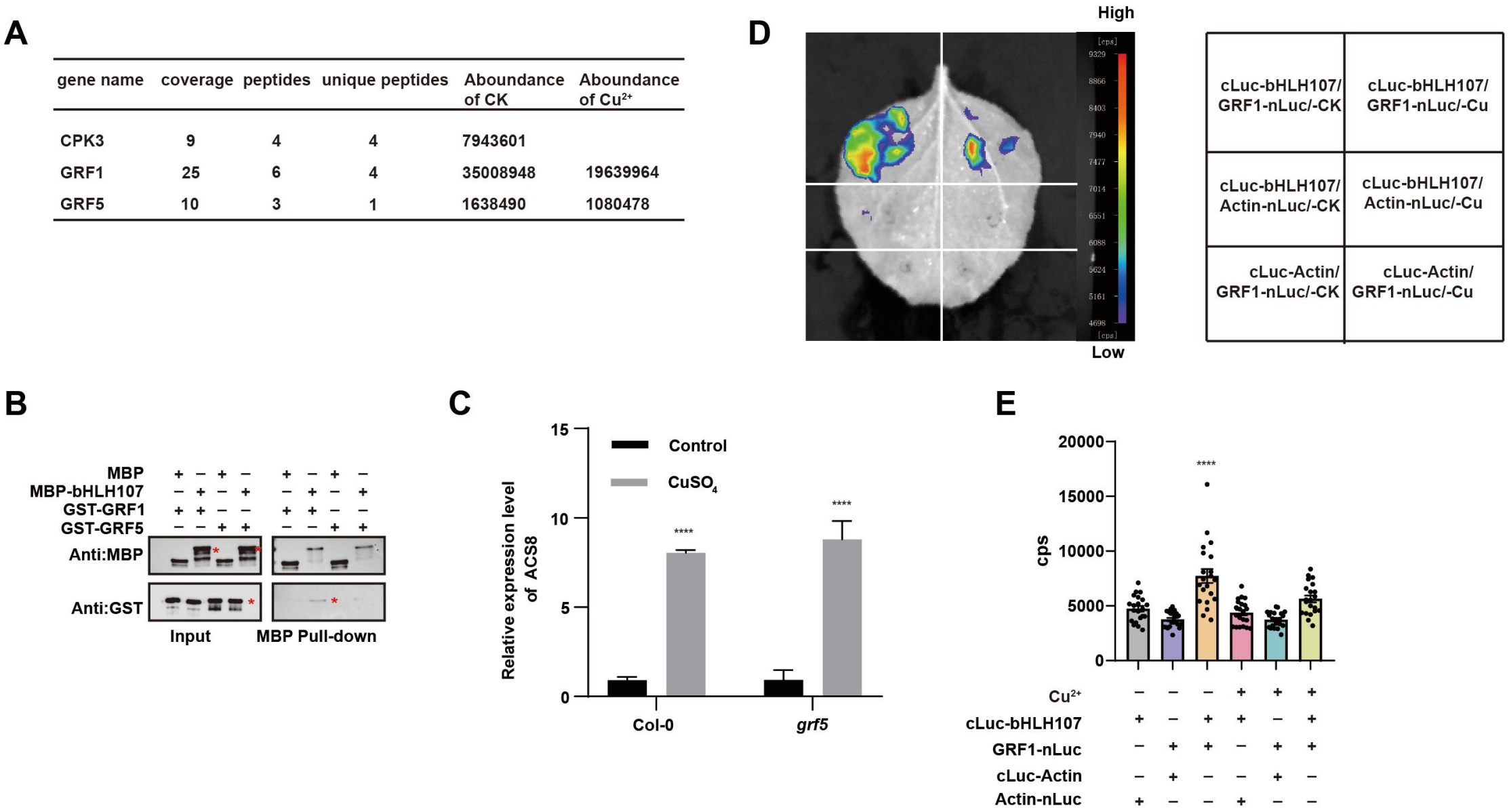

gene name	coverage	peptides	unique peptides	Aboundance of CK	Aboundance of Cu^{2+}
CPK3	9	4	4	7943601	
GRF1	25	6	4	35008948	19639964
GRF5	10	3	1	1638490	1080478

Supplementary Figure 1. Screening of 14-3-3 proteins that interacted with bHLH107

(A) Table for the 14-3-3 proteins of Fig 1 in IP-MS.

(B) Pull down assay showing that GRF1 could direct interact with bHLH107, MBP protein was used as the bait protein for the negative control.

(C) Relative *ACS8* expression in wild-type Col-0 and *grf5*, the samples was harvested at 2 h after treated with or without $CuSO_4$. The expression levels were normalized to those of *Actin2* (*At3g18780*). The error bars represent the means ± SEMs (n = 3). Asterisks indicate significant differences compared with the control of Col-0 (two-way ANOVA)

(D-E) LCA assay was performed to analyze the interaction strength between GRF1 and bHLH107.

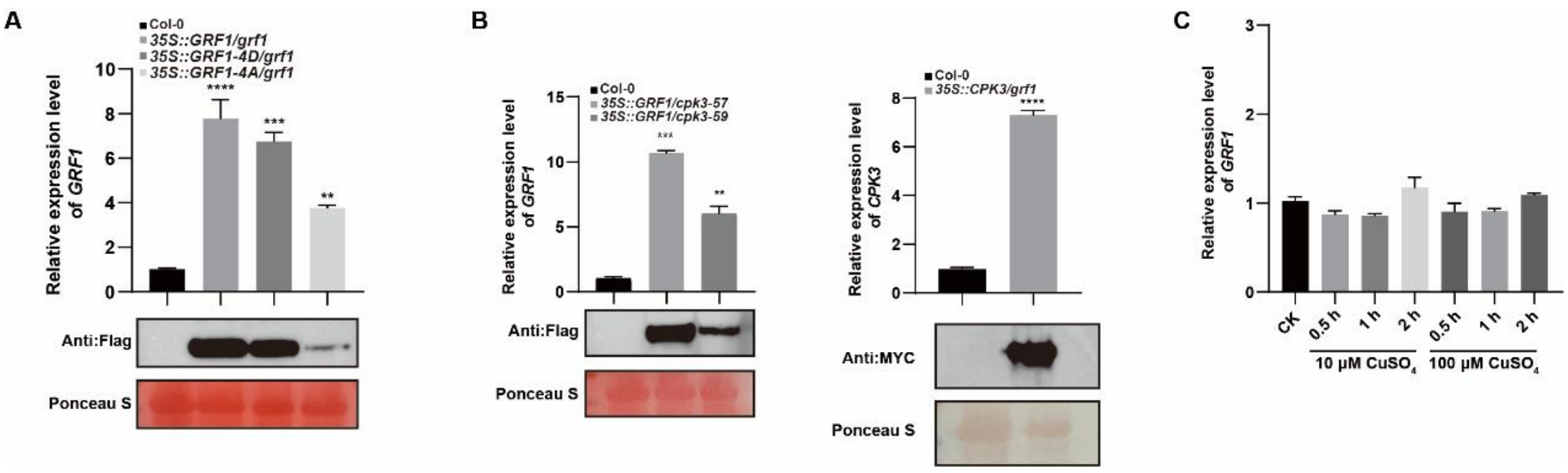

Supplementary Figure 2. Identification of transgenic lines at RNA and protein levels

(A-B) Molecular identification of transgenic lines: RNA (A) and protein (B) level analysis. The error bars represent the means ± SEMs (n ≥ 2). Asterisks indicate significant differences compared with Col-0 (one-way ANOVA). Ponceau S staining was used to quantify the protein loading amount.

(C) Relative expression level of GRF1 in Col-0. The samples were collected after treatment with 10 μM and 100 μM $CuSO_4$ for 0 h, 0.5 h, 1 h, and 2 h, the error bars represent the means ± SEMs (n = 3). Asterisks indicate significant differences compared with the control of Col-0 (one-way ANOVA).

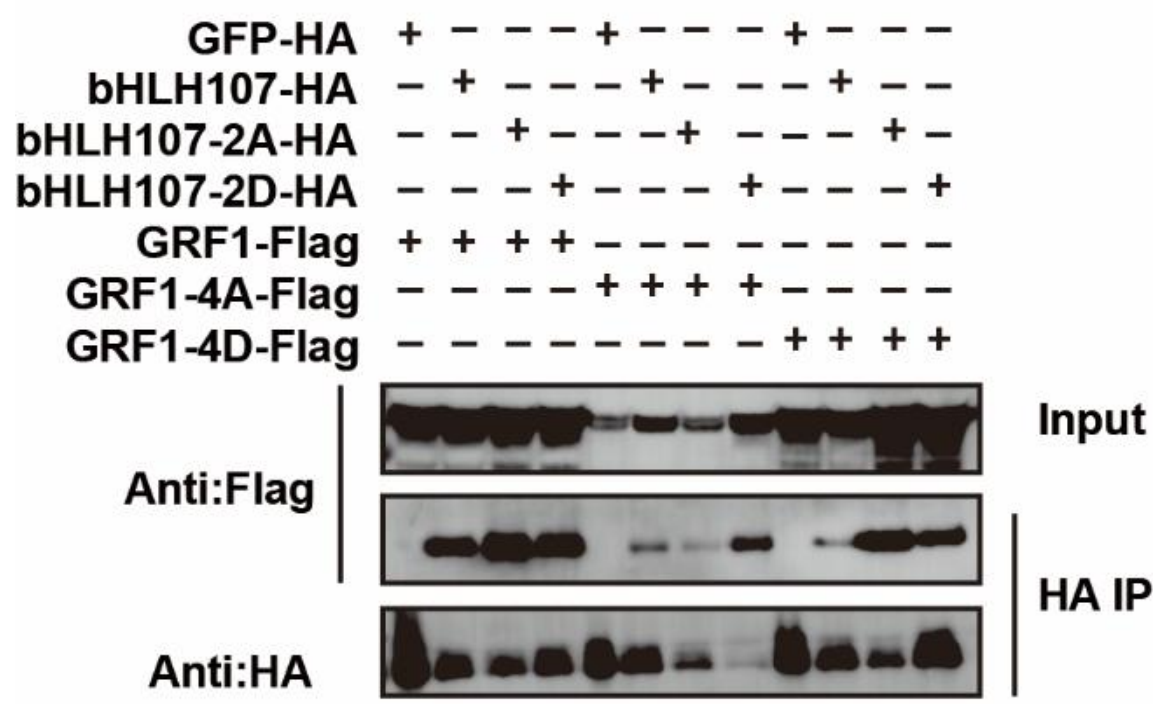

Supplementary Figure 3. Co-IP assays showing that bHLH107, bHLH107-2A and bHLH107-2D interacted with GRF1, GRF1-4A and GRF1-4D.

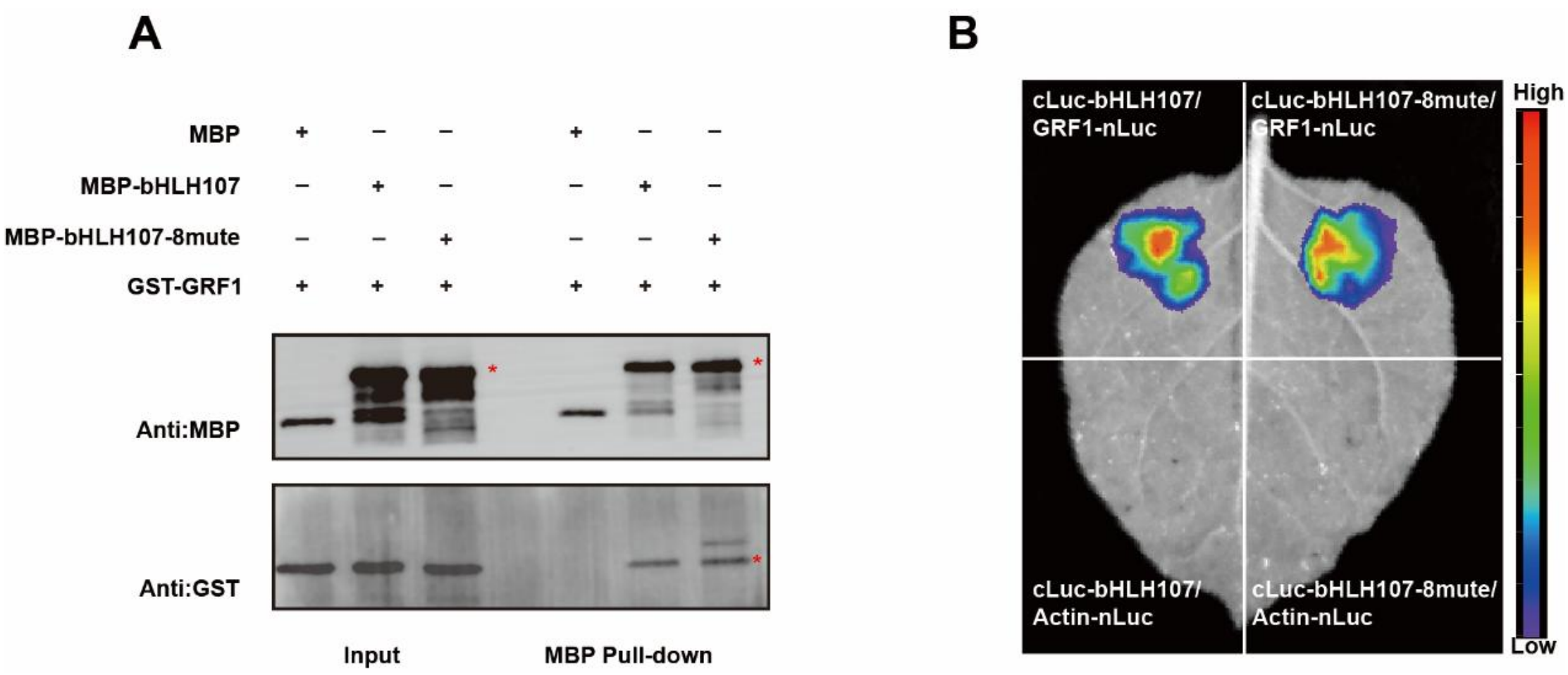

Supplementary Figure 4. Knocking out RINS and KLLS of bHLH107 did not affect the interaction between GRF1 and bHLH107

(A-B) pull-down (A) and LCA (B) assays showing that knocking out two motifs RINS and KLLS of bHLH107 (bHLH107-8mute) did not affect the interaction between GRF1 and bHLH107. The methods used were previously described.

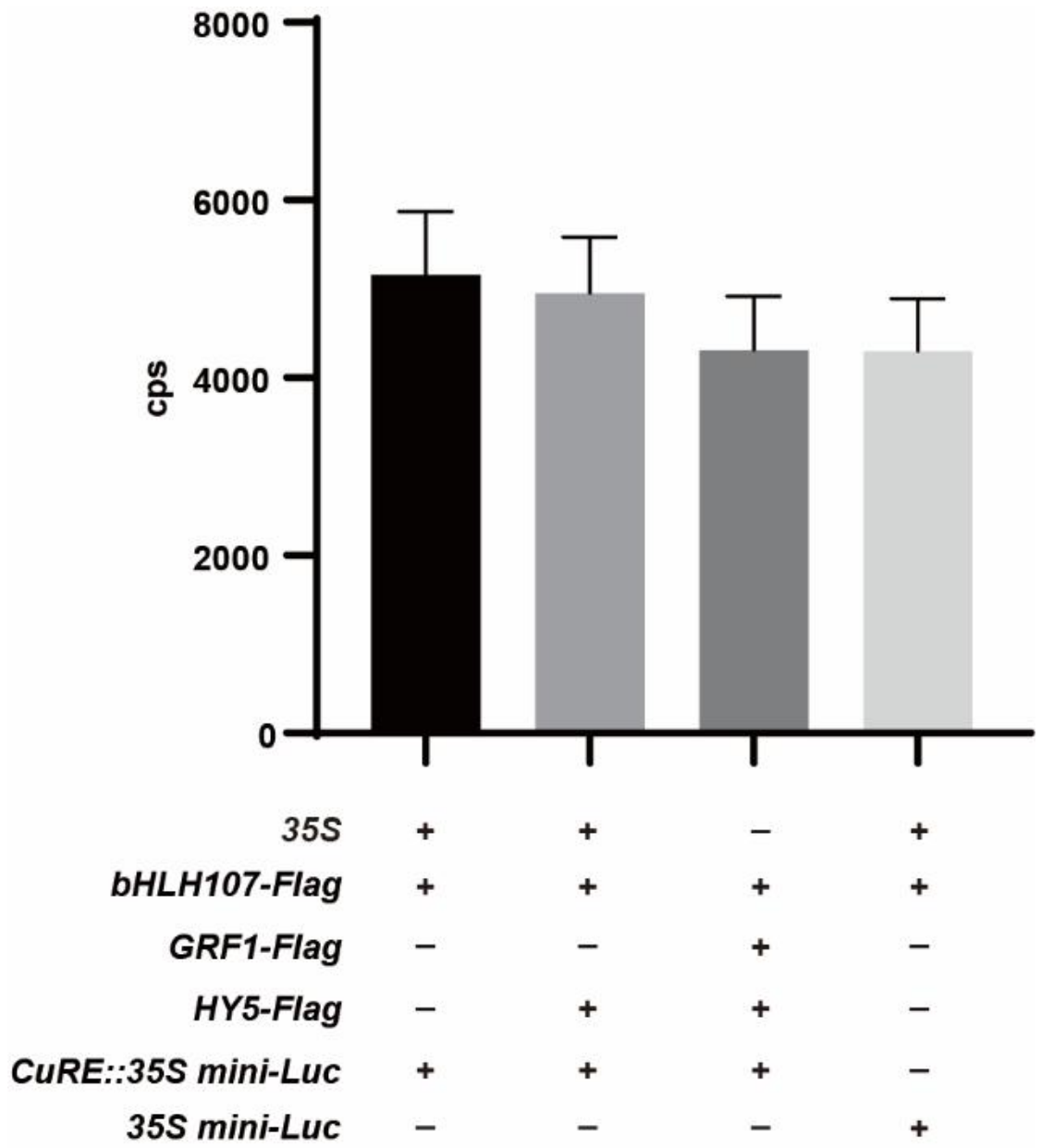

Supplementary Figure 5. GRF1 fails to inhibit the transactivation activity of bHLH107 through HY5 modulation

Relative luciferase activity of *CuRE-35S mini:Luc*, means ± SEMs; n = 12;

Table S1. Primers used in this study.

Primer name	Sequence (5'-3')
Salk018664-LP	TATTTATTGGGCCTATTCGGG
Salk018664-RP	GAAGTTCAACGCAAGACCAAG
LBb1.3	ATTTTGCCGATTTCGGAAC
Genomic-GRF1-Flag-F	atgaccatgattacgaattcAATTATGAATATTTTGATTATT
Genomic-GRF1-Flag-R	cccgggtaccgagctcGGATTGTTGCTCGTCAGCGGGT
CUP2*2-F	CCAAAAGAAGAAGAAAAACCAAAAGAAGAAGAAAAA
CUP2*2-R	TTTTTCTTCTTCTTTTGGTTTTTCTTCTTCTTTTGG
CUP2*2-F-B	CCAAAAGAAGAAGAAAAACCAAAAGAAGAAGAAAAA
CPK3-35SMYC-EcoRI-F	gggggcaatgagatgaattcATGGGCCACAGACACAGCAAGT
CPK3-35SMYC-SacI-R	cccgggtaccgagctcGGGAAATTCGGATCCCTAAGCG
GRF1-35SFLAG-F	gggggcaatgagatgaattcATGGCGACACCAGGAGCTTCCT
GRF1-35SFLAG-R	cccgggtaccgagctcGGATTGTTGCTCGTCAGCGGGT
GRF1-72A-F	gcttcgtggagaatcattGcatcgatcgaacaaaaggag
GRF1-72A-R	ctccttttgttcgatcgatgCaatgattctccacgaagc
GRF1-72D-F	gcttcgtggagaatcattGATtcgatcgaacaaaaggag
GRF1-72D-R	ctccttttgttcgatcgaATCaatgattctccacgaagc
GRF1-88A-F	ggtaacgatgaccacgttGcgttgatccgtgactacag
GRF1-88A-R	ctgtagtcacggatcaacgCaacgtggtcatcgttacc
GRF1-88D-F	ggtaacgatgaccacgttGATttgatccgtgactacag
GRF1-88D-R	ctgtagtcacggatcaaATCaacgtggtcatcgttacc
GRF1-125A-F	gctgctgcttctggagatGcgaaggtgttttatctgaag
GRF1-125A-R	cttcagataaaacaccttcgCatctccagaagcagcagc
GRF1-125D-F	gctgctgcttctggagatGATaaggtgttttatctgaag
GRF1-125D-R	cttcagataaaacaccttATCatctccagaagcagcagc
GRF1-156A-F	aaagatgctgctgaacatGctcttactgcttacaaagctg
GRF1-156A-R	cagctttgtaagcagtaagagCatgttcagcagcatcttt
GRF1-156D-F	aaagatgctgctgaacatGAtcttactgcttacaaagctg

GRF1-156D-R	cagctttgtaagcagtaagATCatgttcagcagcatcttt
NE/CE-GRF1-F	ggcgcgccactagtggatccATGGCGACACCAGGAGCTTCCT
NE/CE-GRF1-R	gcggtaccctcgaggtcgacGGATTGTTGCTCGTCAGCG
NE/CE-CPK3-F	ggcgcgccactagtggatccATGGGCCACAGACACAGCAAGT
NE/CE-CPK3-R	gcggtaccctcgaggtcgacCATTCTGCGTCGGTTTGGCACC
NE/CE-bHLH107-F	ggcgcgccactagtggatccATGCAGCCAGAGGTTTCAGATC
NE/CE-bHLH107-R	gcggtaccctcgaggtcgacGACCATTATGATGTGATCC
GRF1-GST-F	cgcgtggatccccggaattcATGGCGACACCAGGAGCTTCCT
GRF1-GST-R	atgcggccgctcgagtcgacTTAGGATTGTTGCTCGTCAGCG
bHLH107-MBP-F	ggatttcagaattcggatccATGCAGCCAGAGGTTTCAGATC
bHLH107-MBP-R	cgacggccagtgccaagcttTCAGACCATTATGATGTGATCC
CUP2-35Smini-Luc-F	CGGGATCCaagaagaaaaaCGCAAGACCCTTCC
CUP2-35Smini-Luc-R	CGGGATCCGGGACTGACCTACCCGGGG
GRF5-ECOR1-GST-F	CCGCGTGGATCCCCGGAATTCATGTCTTCTGATTCGTCCCGG
GRF5-SAL1-GST-R	ATGCGGCCGCTCGAGTCGACTCACTGCGAAGGTGGTGGTTG
GRF5-LP	AATTCCATTTGGGGAACATTC
GRF5-RP	AGACTGGGGCTTGCTCTTAAC
QRT-genicGRF1-F	ggtgattatcataggtacttggctgag
QRT-genicGRF1-R	gcacgatctggagaattgagaatctc
QRT-GRF1-F	gattctcaattctccagatcgtgcttg
QRT-GRF1-R	gtgctgcttctttgatgtcatcag
QRT-CPK3-F	GACAATGACAACAGCGGCTACATAAC
QRT-CPK3-R	CATTCTGCGTCGGTTTGGCAC
QRT-ACS8-F	CCTTCCTTCCTTCAAGAATGC
QRT-ACS8-R	GAGAGTCTCGTTAGCCGGAGT
QRT-AtAcin2-F	GGTAACATTGTGCTCAGTGGTGG
QRT-AtAcin2-R	GGTGCAACGACCTTAATCTTCAT
